# Gas hydrate versus seabed morphology offshore Lebu (Chilean margin)

**DOI:** 10.1038/s41598-020-78958-z

**Published:** 2020-12-14

**Authors:** Iván Vargas-Cordero, Umberta Tinivella, Lucía Villar-Muñoz, Joaquim P. Bento, Carolina Cárcamo, Diego López-Acevedo, Francisco Fernandoy, Alessandra Rivero, Marion San Juan

**Affiliations:** 1 Valparaiso, Chile; 2 Istituto Nazionale di Oceanografia e di Geofisica Sperimentale - OGS, 34010 Trieste, Italy; 3 Centro de Investigación y Formación San Ignacio de Huinay, Hualaihué, Chile; 4 Departamento de Geofísica, Facultad de Ciencias Físicas y Matemáticas, Universidad de Chile, Santiago, Chile; 5 Escuela de Ciencias del Mar, Pontificia Universidad Católica de Valparaíso, Valparaíso, Chile; 6 Universidad Andres Bello, Viña del Mar, Chile; 7 Universidad de Chile, Santiago, Chile

**Keywords:** Climate sciences, Environmental sciences, Natural hazards, Solid Earth sciences

## Abstract

Gas-hydrate occurrences along the Chilean margin have been widely documented, but the processes associated with fluid escapes caused by the dissociation of gas hydrates are still unknown. We report a seabed morphology growth related to fluid migration offshore Lebu associated with mud cones by analysing oxygen and deuterium stable water isotopes in pore water, bathymetric, biological and sedimentological data. A relief was observed at − 127 m water depth with five peaks. Enrichment values of δ^18^O (0.0–1.8‰) and δD (0.0–5.6‰) evidenced past hydrate melting. The orientation of the relief could be associated with faults and fractures, which constitute pathways for fluid migration. The benthic foraminifera observed can be associated with cold seep areas. We model that the mud cones correspond to mud growing processes related to past gas-hydrate dissociation. The integration of (i) the seismic data analysis performed in the surrounding area, (ii) the orientation of our studied relief, (iii) the infaunal foraminifera observed, (iv) the grain size and (v) the total organic matter and isotope values revealed that this area was formerly characterised by the presence of gas hydrates. Hence, this part of the Chilean margin represents a suitable area for investigating fluid-migration processes.

## Introduction

Along continental margins, morphological features associated with fluid escapes such as mud volcanoes, mud mounds, pockmarks, seeps, precipitates—carbonates and hydrates-, piping/rills and brine pools have been reported worldwide (e.g.^1–7^). Fluid escape morphologies related to gas escape include a) seafloor depressions as pockmarks and b) seafloor mud growing as mud volcanoes, mud cones, mud diapirs and mounds. Although numerous fluid escape features have been identified in diverse tectonic settings, there is still little information on the distinct processes and factors that form and control them. Some factors are evident, such as the chemistry of expelled fluids, and the fluid flow rates and duration. As a first approach, the accumulation of sediment in the form of mud growing, such as mud volcanoes, has been related to the rapid expulsion of fluids under overpressure. Pockmarks are associated with gas expulsion in near-surface sediments, whereas slow seepage promotes lithification of the seafloor through precipitation of a variety of mineral species (e.g.^8–11^).

Some authors describe mud growing morphologies as mud volcanoes and mounds when they are isolated, and as mud cones and mud diapirs when they are aligned in space^12,13^. Others indicate that when the extruded sedimentary material is responsible for the seafloor morphology, it can be described as mud volcanoes and mud cones^14^, whereas when the fluidised material is not expelled, it can be defined as mud diapir^1^. Fluid escapes can be formed mainly by microbial and thermogenic methane gas and water^15,16^. This gas can give place to gas-hydrate formation in marine sediments if pressure and temperature conditions are adequate^17^; in this condition, the gas is trapped in a lattice of water molecules.

Along continental margins, gas hydrates occur naturally within the Gas Hydrate Stability Zone (GHSZ), mostly at sub-marine sediment depths greater than 300–500 m with low temperature, high pressure and adequate amounts of sedimentary organic carbon (2–3.5%), where enough methane is present^18,19^. Moreover, salinity, gas composition, geological structure, fluid migration, and pore space of marine sediments influence gas-hydrate formation^18–20^. Gas-hydrate occurrences along the Chilean margin are distributed from 33 to 57°S as widely reported in literature^21–31^. On the other hand, only a few studies have documented fluid escapes related to gas-hydrate dissociation through faults and fractures (e.g.^32,33^).

Identifying areas where gas-hydrate dissociation occurs is essential. The presence of methane, a potent greenhouse gas^34^, in zones of shallow fluid escapes could may contribute to (a) an increase in surface temperature in favour of global warming, (b) change in the physic-chemical conditions of seawater, (c) affect the marine microfaunal diversity pattern, and (d) affect the nucleation and the rupture propagation of earthquakes, related to slides and tsunamis^35–37^.

The most common techniques used to identify gas-hydrate dissociation zones are biological, geochemical and geophysical analyses techniques. Biological indicators such as benthic foraminifera, bivalve and microbial communities, were related to fluid escapes^38–42^. For example, foraminiferal taxa reported worldwide, including *Uvigerina *sp.,* Bolivina *sp.,* Chilostomella *sp.,* Globobulimina *sp.,* Quinqueloculina* sp. and *Nonionella *sp., were associated to cold seep occurrences and the presence of methane^43–45^. These organisms can live in organic-rich and reducing environments, where food availability is high^43–45^. In different regions including the Chilean coast^46–48^, reported several cases of enriched stable water isotope values (as measured from pore water in marine sediments) linked to gas-hydrate dissociation. The reported values for δ^18^O and δD reached up to 3‰ and 10‰, respectively. Geophysical studies documented seabed morphologies in relation to fluid leakages, using acoustic data and high-resolution images^4,49,50^.

In south-central Chile, the simultaneous interpretation of direct and indirect geophysical data is essential to determine the active structural domain located in the vicinity of the Arauco basin^51,52^. These geophysical data made it possible to recognise the exchange of subsidence and uplift events responsible for the accretion and erosion of the prism^53,54^. The geophysical data interpretation indicated that the sedimentary sequences are quite complex and distinguished by marine and continental deposit cycles. These environments are compatible with the presence of gas and carbon deposits detected in the past^55,56^.

This study aims to understand whether past gas hydrate dissociation could be related to the morphological growth observed on the Lebu coast (Chilean margin). To address our objective, we used direct and indirect evidence of the presence of gas hydrate dissociation, including geophysical, biological and geological sampling as well as physical–chemical and geochemical analyses.

## Materials and methods

The data, methods and techniques used in this work were based on a multi- and inter-disciplinary approach to characterize the morphology and the fluid escapes; field and laboratory data, theory and modelling approaches, were used. The approach included bathymetric data processing and sedimentological, physical–chemical, geochemical and biological analyses of seawater and marine sediment samples.

### Data sampling

**Figure 1:**
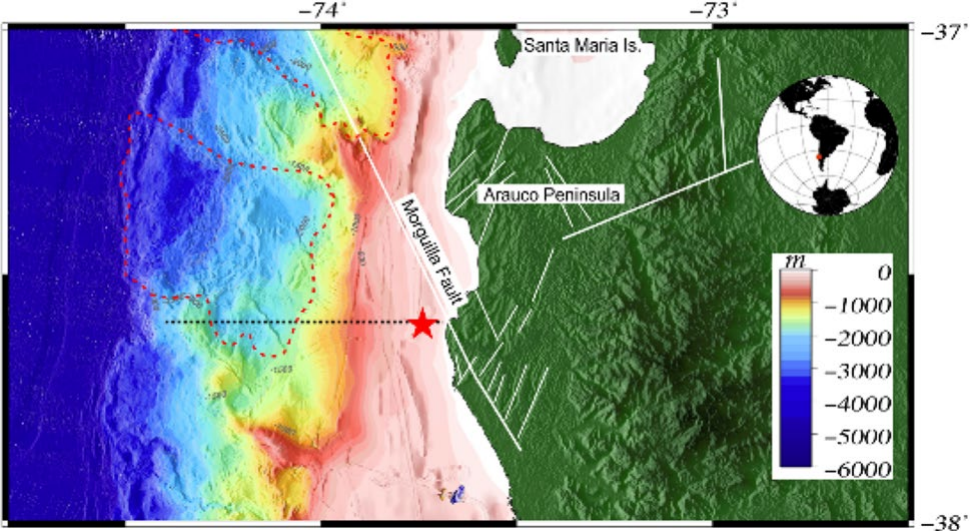
Location map of the studied area. The red star shows corer recovery and bathymetric surveys. The black dashed line shows the bathymetric profile used in Fig. 8, the white solid lines indicate faults and fractures described by several authors^53,92,93^ and the red dashed lines show embayments related to ancient slides reported by^92^.

In the framework of the project entitled “Identification and quantification of gas emanations associated with gas hydrates (FONDECYT 11140214)”, sedimentological, geochemical and bathymetric studies offshore Lebu were performed (Fig. 1). In 2016 and in 2017, two oceanographic cruises onboard R/V Kay Kay II were carried out to collect bathymetric data, seawater samples and marine sediments.

**Figure 2:**
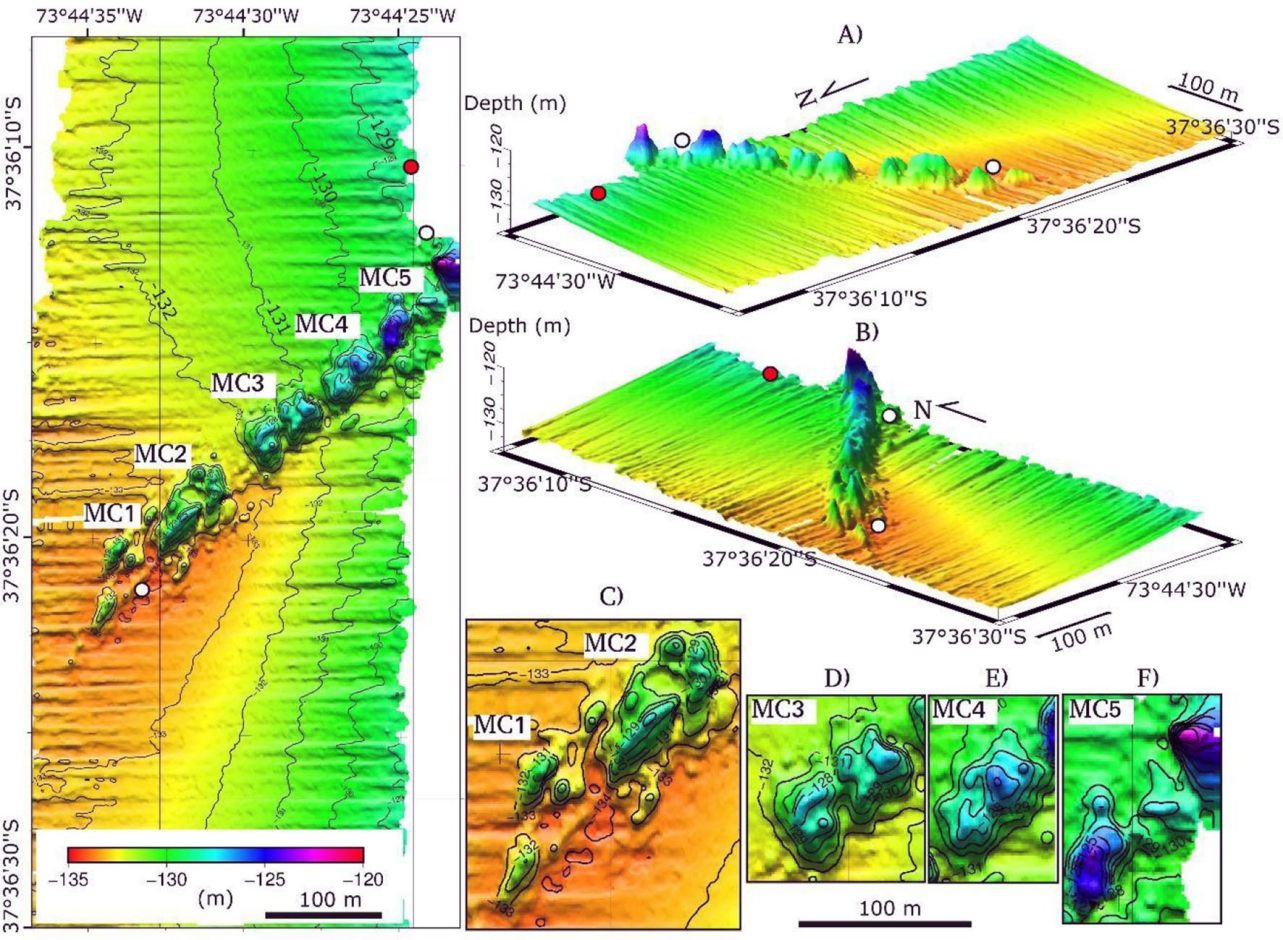
Bathymetric map indicating location corer GC-02 (red circle), water samples (white circles) and identified mud cones (MC1, MC2, MC3, MC4 and MC5). In (**A**) and (**B**) 3D images with orientation NW and SW respectively, while in (**C**), (**D**), (**E**) and (**F**) zooms for mud cones are reported.

Marine sediment samples were collected about 127 m below the sea level (mbsl), using a gravity corer (GC-02; 9 cm diameter) that drilled 240 cm into the seabed. The corer was deplyed at the northern of the positive relief (at a distance of approximately 100 m; 73°44′ 25″ W/37°36′ 10″ S; Fig. 2), and the sediment-core sample was divided into four sections of 60 cm long. Each sediment-core section was frozen on board for later analysis.

Water samples were collected using Niskin bottles at 0 m, 10 m, 20 m, and 50 m below sea surface, and above the seafloor. Temperature, conductivity, dissolved oxygen, and pH, were determined using the multiparameter Meter (IP67, model 8602). These parameters were measured at the two ends of the identified lineament (Fig. 2). The measurements were obtained near the relief to evaluate the relationship between marine sediments and the water column in the presence of ancient gas hydrates.

Bathymetric data were acquired using a multibeam Reson SeaBat 7125 echosounder (400 kHz, 0.5° × 1°). Sound velocity data were acquired using a SVP90 probe, and an AML Oceanographic Model Minos X sound velocity profiler. Preliminary processing was performed on board using a PDS2000 commercial software allowing real-time bathymetric data processing using the SVP90, the AML information, and the ship motion (pitch, roll, yaw and heave).

The PDS2000 software used real-time information from other sensors to calculate the Total Propagated Error (TPE), specifically the vertical base reduction, and total heave and depth. Each sounding was filtered by setting the International Hydrographic Organization (IHO) Order 2.

### Methods

The acoustic data processing was performed using the open-source MB-System libraries^57^, to model the bathymetry of the region, with tide and scattering effects attenuated. Bathymetric grids were created with the nearest neighbour interpolation algorithm, using the open-source software Generic Mapping Tools (GMT;^58^). The algorithm constructs regularly gridded values, in which each node corresponds to the weighted mean of the samples around a search circle of 5 m. The selected grid was configured with a spatial resolution of 1 m. A squared median filter of 5 m was applied to smooth the grid. Finally, the bathymetric accuracy was off 0.2% in-depth. These values are in agreement with the accuracy values reported by other authors^59^. The standard deviation for depth ranged from 0.09 m to 0.27 m for 128.59–133.87 m of depth.

Grain size analysis was performed by sieving method where sediments passed (by agitation) through a range of different-size meshes. Fifty grams of sediments were sieved using the following mesh sizes: 60, 80, 120 and 230. Alternatively, the pipette method was adopted to separate clay and silt fractions by selecting 15 g of mud sample. Statistical parameters were calculated in agreement with reported formulas^60–62^.

Surface to seafloor water physical–chemical properties of temperature, pH, salinity, and dissolved oxygen to near the positive relief, were obtained using the multiparameter Meter. Temperature was measured in Celsius degrees, with an accuracy of ± 0.5 °C, and pH was directly related to the concentration ratio of hydrogen ions [H +] and hydroxyl [OH]^63^ with an accuracy of ± 0.1. Salinity was obtained from conductivity, with an accuracy of ± 0.1, dependent on the number of dissolved ions per unit volume and on the mobility of the ions. Dissolved oxygen was measured in % and in mg/L, with an accuracy of ± 3%.

The sediment-core sample was cut in sections of 10 cm long, and physical–chemical parameters were measured, including water content (W in %), porosity (Φ in %), the content of solid material per unit volume, expressed as apparent density (ρ in g/cm^3^;^64^) and Total Organic Matter (TOM in %). Sediment samples were dried in a forced-air oven at 60 °C for 36 h and in a desiccator for 30 min.

TOM content was measured by gravimetric determination of weight loss through the loss-on-ignition method^65,66^. Two grams of dry sediment sample were calcined in a muffle at 500 °C for 5 h and placed in a desiccator for 30 min until stable weight was obtained, in order to reduce the associated error.

For foraminiferal sampling and identification, the sediment-core sample was cut into sections of 15 cm of which 50 g of material was extracted. The material was washed, dried, and sieved using 120 and 230 diameter sieves. Specimens were placed in Petri dishes and observed under binocular magnification. General morphological features were characterized using the Atlas of Benthic Foraminifera^67^, and the genus were identified based on the study of Chilean material (e.g.^68^).

Pore water from the sediment-core samples was extracted using an ACME lysimeter (0.2 μm) to analyse oxygen and deuterium stable water isotopes. Pore water extraction procedure involved cutting the sediment core in sections of 5 cm long, centrifugation, and pore water extraction using Rhizon MOM with pore sizes ranging from 0.12 µm to 0.18 µm. Stable water isotope was determined by Cavity Ring-Down Spectroscopy (CRDS) method.

Deuterium water isotope and oxygen content were evaluated using in-house standards LIMS^69^, normalised to the VSMOW-SLAP scale, and values were reported as δ-values for deuterium (δD) and oxygen (δ^18^O). Each sample was measured at least twice on different days. For each measurement, samples were analysed for five consecutive times. Results were accepted if the standard deviation of each single run (composed of five repetitions) was < 1‰ for δD and < 0.1‰ for δ^18^O. The accepted stable water isotope value of a sample was the mean of (at least) 2 different valid measurements within the range of the explained standard deviation.

## Results

### Morphology

Bathymetric data revealed a positive relief with orientation N55°E at 127 mbsl. The relief showed a mean elevation of about 6 m above the seafloor, an extension of 410 m length and a width of 50 m, corresponding to an area of 14,640 m^2^ (Fig. 2). The relief was composed of five mud growing. They were aligned (see Introduction;^12,13^), thus defined as mud cones (see Fig. 2 MC1, MC2, MC3, MC4, MC5). They were characterized by peaks ranging from 3 to 9 m height, with MC5 reaching the maximum value of 9 m in height. MC1 showed 2 elongated shape cones of about 40 m length and 20 m width, with steep and smooth sides. MC2 displayed elongated and circular shape cones of about 80 m length and 50 m width, mainly exhibiting steep sides. MC3 and MC4 presented rectangular shapes cones of 60 m length and 50 m width, and their morphologies varied from smooth to steep sides. MC5 showed elongated and circular shape cones with smooth and steep sides.

### Physical–chemical parameters

**Figure 3:**
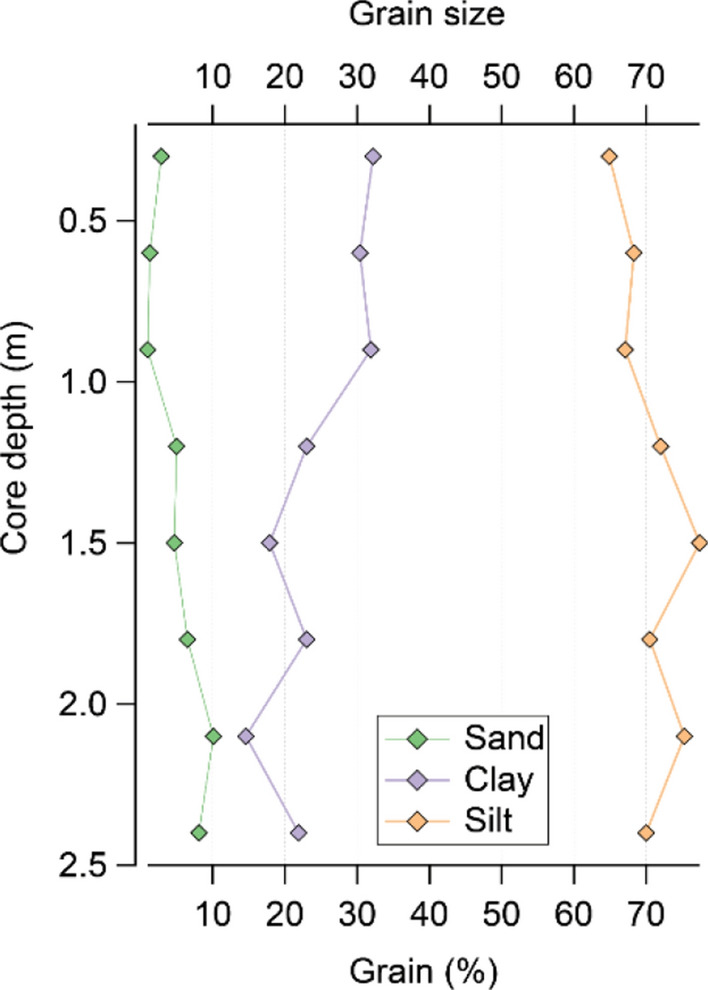
Grain size distribution in marine sediments (corer GC02).

Grain size analysis showed constant values with depth. The mean grain size corresponded to sandy mud textural group, characteristic to mud cones. Silt comprised 60% of the total sediment-sample volume (Fig. 3).

**Figure 4:**
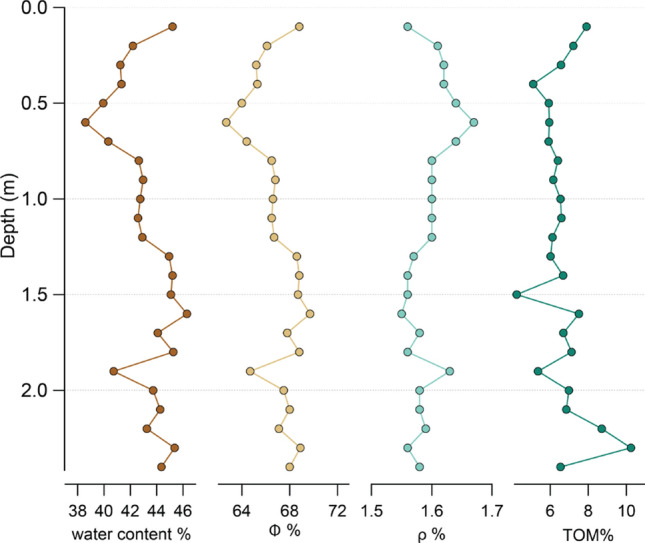
Physical–chemical parameters distribution in marine sediments (corer GC02).

A slight variation of water content (W), ranging from 38.6 to 46.3% (mean 43.1%), porosity (ɸ), ranging from 62.7 to 69.7% (mean 66.9%) and an apparent density (ρ), ranging from 1.5 to 1.7 g/cm^3^ (mean 1.6 g/cm^3^), were detected (Table 1). TOM values showed a variable trend with a maximum value of 8.7% of total volume located at 2.2 mbsl, while the minimum value of 5.1% of total volume detected at 0.4 mbsl (Fig. 4). An opposite trend distribution was observed between porosity and apparent density.

**Table 1:** Physical–chemical parameter distribution in marine sediments.

Depth (m)	W (%)	ɸ (%)	Ρ (g/cm^3^)	TOM (%)
0.1	45.2	68.8	1.56	7.9
0.2	42.2	66.1	1.61	7.2
0.3	41.2	65.2	1.62	6.6
0.4	41.3	65.3	1.62	5.1
0.5	39.9	64.0	1.64	5.9
0.6	38.6	62.7	1.67	6.0
0.7	40.3	64.4	1.64	5.9
0.8	42.7	66.5	1.60	6.4
0.9	43.0	66.8	1.60	6.2
1	42.8	66.6	1.60	6.5
1.1	42.6	66.5	1.60	6.6
1.2	42.9	66.7	1.60	6.1
1.3	45.0	68.6	1.57	6.0
1.4	45.2	68.8	1.56	6.7
1.5	45.1	68.7	1.56	4.2
1.6	46.3	69.7	1.55	7.5
1.7	44.1	67.8	1.58	6.7
1.8	45.3	68.8	1.56	7.1
1.9	40.7	64.7	1.63	5.4
2	43.7	67.5	1.58	7.0
2.1	44.3	68.0	1.58	6.8
2.2	43.3	67.1	1.59	8.7
2.3	45.4	68.9	1.56	6.9
2.4	44.4	68.0	1.58	6.5
Mean	43.1	66.9	1.59	6.5
Minimum	38.6	62.7	1.55	4.2
Maximum	46.3	69.7	1.67	8.7

### Isotope analysis

**Figure 5:**
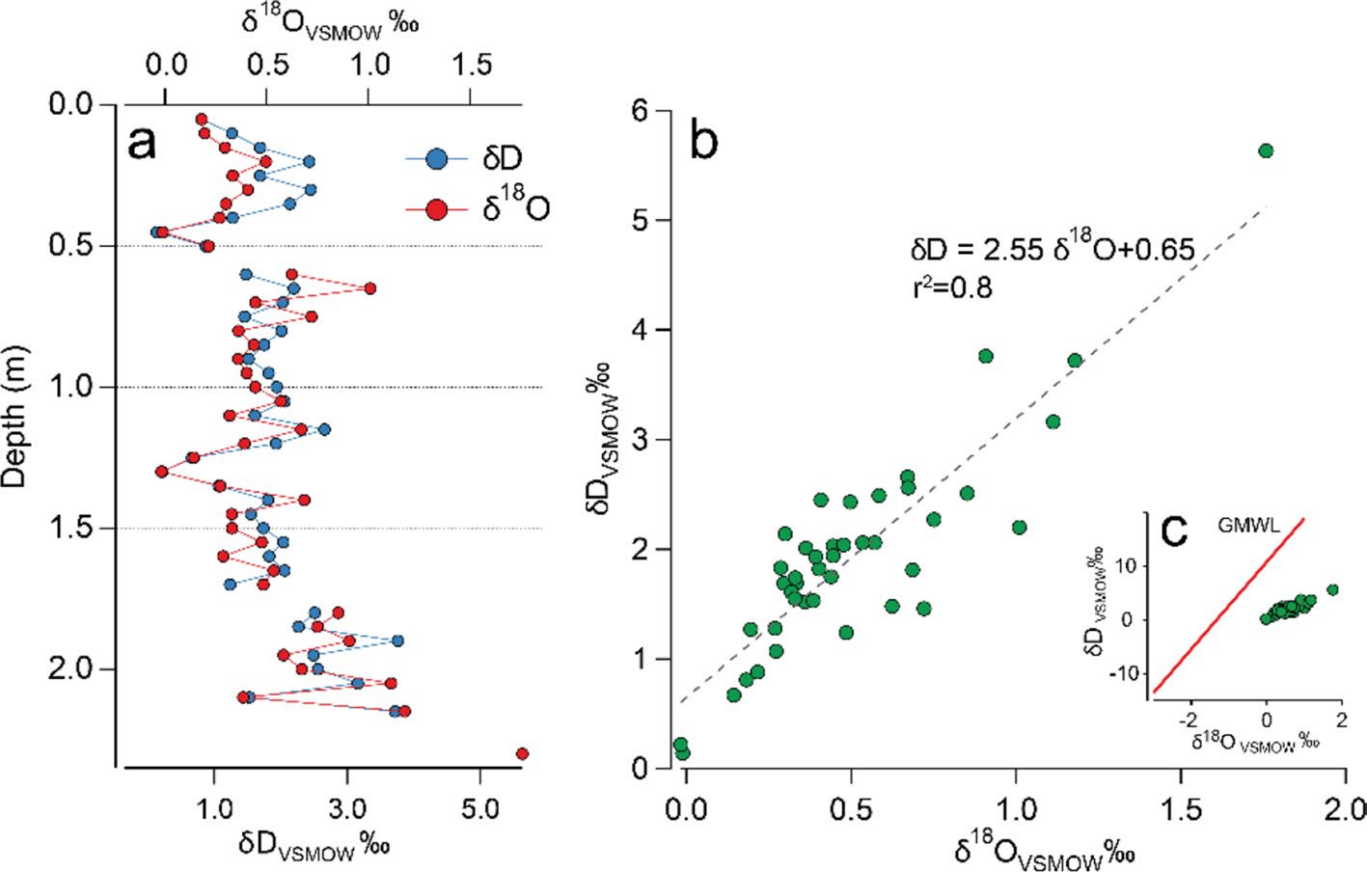
Oxygen (δ^18^O) and deuterium (δD) stable water isotope distribution in sediment from: (**a**) Depth profile of the corer; (**b**) Cross-isotope linear regression of pore water samples and (**c**) Cross-isotope relationship of pore water samples against the global meteoric water (GMWL).

Pore stable water isotope analysis of the sediment-core samples showed positive values ranging from 0.0 up to 1.8‰ for δ^18^O and 5.6‰ for δD (Fig. 5). Stable water isotope δ-values were close to 0.0 at the sediment–seawater interface, and the values increased with depth of the sediment-core samples (i.e. enrichment), with low variability (δ^18^O, 0.33 Std. Dev.; δD 0.95 Std. Dev.). No negative δ-value was observed in any of the sediment-core samples analysed.

### Biological communities

**Figure 6:**
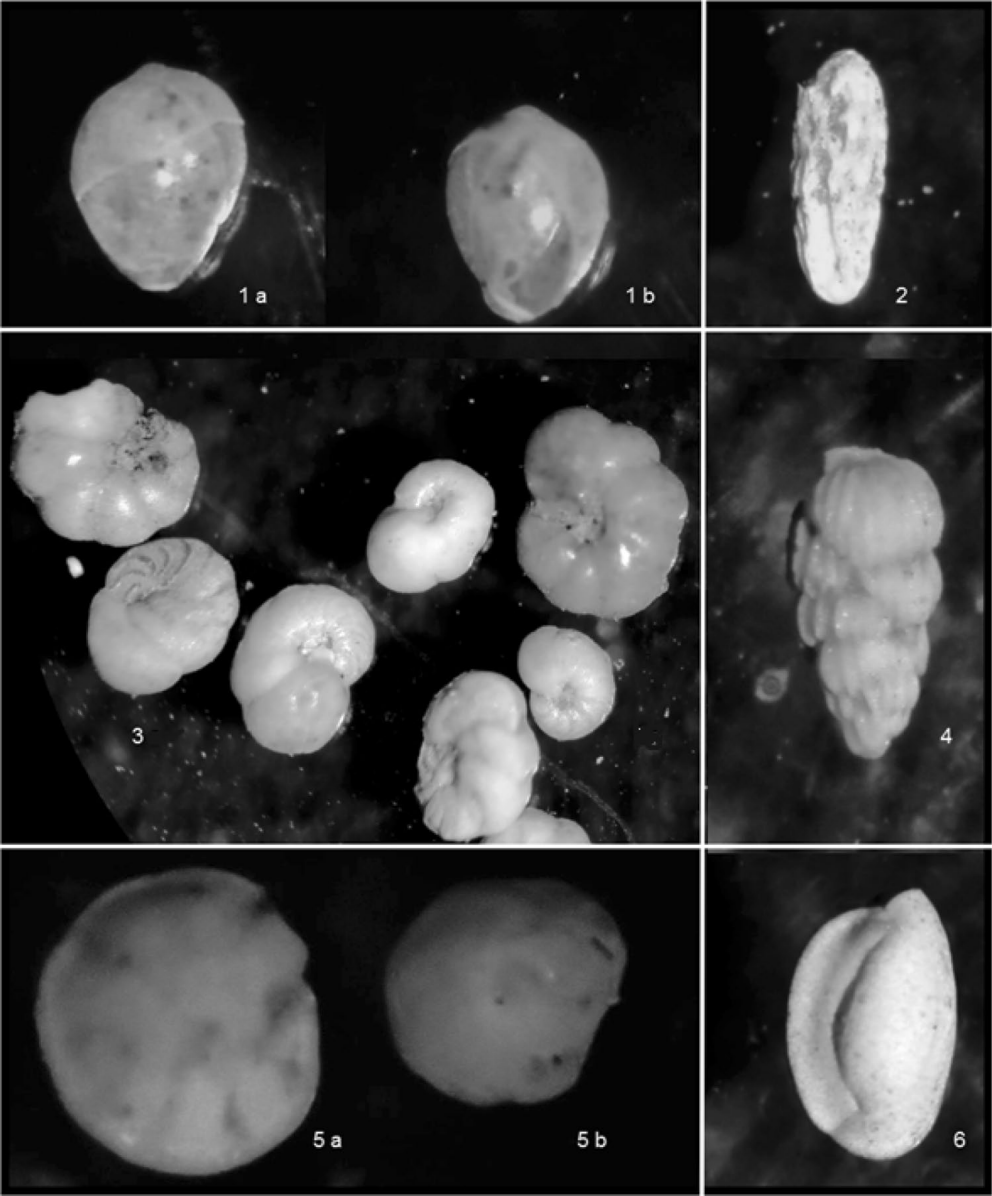
Benthic foraminifera. In (1a) *Globobulimina *sp., lateral view (10×); (1b) *Globobulimina *sp., lateral view (10×); (2) *Bolivina plicata*., lateral view (5×); (3) *Anomalinoides* spp., lateral view (5×); (4) *Uvigerina peregrina*, lateral view (5×); (5a) *Oridorsalis tener*, lateral view (5×); (5b) *Oridorsalis tener*, lateral view (5×); (6) *Quinqueloculina vulgaris*, lateral view (10×).

Benthic foraminiferal aggregations were uncovered in the top section of the sediment-core samples (0–60 cm), comprising globose and elongated specimens. The following taxa where identified, including opportunistic species: *Globobulimina* sp., *Bolivina plicata*, *Anomalinoides* sp., *Uvigerina peregrina*, *Oridorsalis tener*, and *Quinqueloculina vulgaris* (Fig. 6).

### Water properties

Water column temperature decreases from 14 °C at the surface to 12 °C at 20 mbsl. Salinity and dissolved oxygen content also decreased with depth, from 31 to 33‰, and 60–66.2% were minimum at 0.6 mbsl. pH values ranged from 7.5 to 8.1 from surface to depth.

## Discussion

Stable water isotope composition of pore water provides strong evidence of gas-hydrate dissociation. Heavy oxygen isotope enrichment with depth can be explained only by isotopic fractionation after hydrate dissociation (e.g.^70^). The fractionation process occurs during hydrate formation, which concentrates the heavy isotopes (e.g. δD and δ^18^O) in the hydrate layer, as a result of the dissociation pressure of the ice-lattice Thus, hydrate dissociation yields pore fluids with more positive δ^18^O values by comparison to seawater.

Figure 5a presents the stable water isotope profile of the entire sediment-core sample, showing a clear increase in isotope value with depth, with values close to 0‰ at the seawater-sediment interface to positive values at the bottom of the corer. In addition, observational data at similar latitudes, and modelled surface water stable isotope composition for this ocean region, showed that shallow water tend to present slight negative isotope composition (∼− 0.2‰ to − 0.5‰ δ^18^O;^71,72^), which are related from the transport of Sub Antarctic Waters through the Humboldt Current System along the Chilean coast^73^. Negative isotopic values result mainly from the mixture of oceanic and depleted meltwater from the Antarctic Ice Sheets^74^. In this study, the isotopic trend was indicative of seawater mixing at the top of the sediment-core sample, and of different water source at the bottom of the core. The cross-isotope relationship of our samples shows that the stable water isotope composition of pore water has a strong positive correlation (e.g. simultaneous enrichment of δ^18^O and δD; Fig. 5b). Positive δ^18^O values are associated to clay minerals dewatering, generally related to a robust δD decrease^47^. Our results showed an increase in δD, as was also observed by^75^ in the same relief, agreeing with the hypothesis of past hydrate melting^46,47,76–78^. Note positive values of meteoric waters are negligible, as shown in Fig. 5c, because porous waters deviate from the global meteoric water line.

The infaunal foraminifera, found in the shallower sediment samples (e.g. *Bolivina *sp., *Globobulimina *sp.,* Uvigerina* sp.), may be associated with modern cold seeps. These taxa can metabolize seeping methane, directly or indirectly exploiting the available geochemical energy source^79^, as documented by several authors in cold-seep area (e.g.^44,80,81^), including the continental margins of California, Japan and Mexico^36,43–45,82^. Benthic communities are often found in high organic-content ambient and low oxygen environments, characteristic to cold seep^44,45^. In this study, TOM was 6.5%, suggesting that the organic-carbon stock offshore Lebu promoted the development of benthic foraminifera, as demonstrated by^83^ who found a positive correlation between foraminiferal distribution and at least of 1.5% TOM.

Grain-size data provide crucial information about hydrodynamic conditions; in particular, mud and sand content associated to coastal and beach systems and riverine or deltaic deposits^84^. Recently^85^, indicated that grain size decreases generally with the pore size, and that sediment permeability decreases with decreasing pore size. Consequently, the formation of gas hydrate is limited in low-grain sized sediments, because of reduced flow of gas and water to the sediments. Clay- and silt-particle agglomerates may lead to an increase in both apparent grain size and pore throat size^86^, maintaining the relative permeability of the sediments, which promotes the formation of gas hydrate in silt-rich sediments. The depth trend of the physical–chemical parameters measured in this study (W, ɸ, ρ, TOM), in relation to grain size, permitted to describe the benthic environment offshore Lebu. Our approach was particularly relevant given the relationship between clay and silt content and high TOM values^87^, which are favourable to gas-hydrate formation (e.g.^88,89^).

Seawater temperature, salinity, dissolved oxygen and pH were typical to the study region^75^. Water-column temperature decreased with depth and salinity and dissolved oxygen were inversely proportional^63^. Seawater alkalinity generally assumes pH values between 7.4 and 8.4, as obtained in our sample. Gas phases concentrations were estimated across the continental slope zone off Lebu by^25^, reporting 15% and 0.2% of total volume for hydrates and for free gas, respectively. As pointed out by^90^, the high proportion of gas hydrate amount may be generated by advective flow of dissolved hydrate or gas from depth, Other studies suggest that lateral fluid migration occurs from deep levels through faults and fractures canalising fluids that lead to the formation of mud cones and mud volcanoes (e.g.^32,33^). Several researchers reported faults in the proximity of our study area (Fig. 1), such as the Santa María fault of a similar orientation to the relief document in this study (N55°E;^51,91,92^). Gas accumulations can reach shallow areas because the base of the gas-hydrate stability zone can be very shallow on continental shelves, as indicated by theoretical modelling^25^. Therefore, the integration of (i) the seismic data analysis performed in the surrounding area^25^, (ii) the orientation of our studied relief, (iii) the infaunal foraminifera observed, (iv) the grain size and (v) the TOM and isotope values reported here, suggest that this area was characterised by the presence of gas hydrate.

The gas hydrates dissociation and fluid expulsion in this region may be related to (a) regional uplift occurring since late Pleistocene-Holocene^52,93,94^, (b) deglaciation processes, (c) variation in heat flow caused by landslides, and (d) the vertical variability of the equatorial subsurface water mass (ESSW), transported poleward by the Peru–Chile undercurrent (PCUC) and the El Niño-Southern Oscillation (ENSO). In the Arauco basin, several authors reported a surface uplifting of about 1.5 km during the Middle Pliocene^51,52^. Basal accretion processes may be responsible for the characteristic uplifting of the Arauco region coast and shelf systems^93,95^. The constant uplifting in this area may decrease the hydrostatic pressure of the sediments and dissociate the gas-hydrate layer as reported in other areas worldwide^96–99^. On the other hand, deglaciation processes starting, which started 20,000 years ago in Chile^100–102^, lead to warmer seabed temperatures in shallow waters promoting gas-hydrate dissociation. Off the Arauco Peninsula, ancient landslides (see Fig. 1;^103^) may increase the heat flow as a result of fluid advection area or increased geothermal gradient^29^. Heat-flow rise also promotes hydrate dissociation, as reported in Mocha Island close to our study area^104^.

Finally, in this region^105^, find the subsurface oceanic poleward flow, the so-called PCUC, originated in subtropical regions (6°S). This current flows from surface water to 150 mbsl, transporting the ESSW warm temperatures, high salinity, high nutrients, and low oxygen content along the continental shelf and slope off Chile up to 48°S^73,106^. Essential physical processes that drive the vertical variability of ESSW include mesoscale intrathermocline eddies that propagate westward^105^, and their transport of volume that is significantly correlated with the variability of PCUC transport of water, which in turn is forced by the ENSO equatorial signal^107^. Intra-seasonal coastal trapped waves and Rossby waves result mainly from the teleconnection between the equatorial Pacific and South America, particularly during El Niño events^108–110^, and other high-frequencies coastal internal waves forced locally^111^. Temperature data sampled 28 years apart revealed decadal warming of the middle to the deep-ocean layers close to the study area^112^, which may explain gas-hydrate dissociation in this study^17,113^.

**Figure 7:**
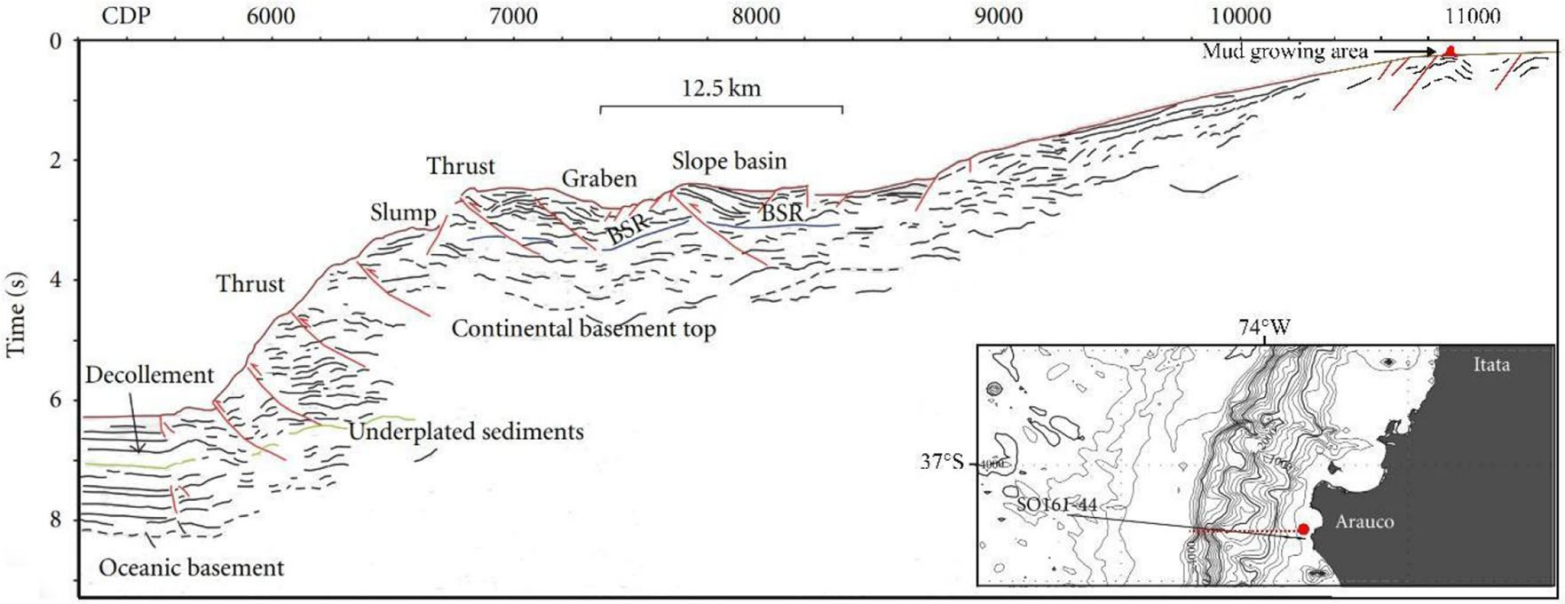
Line drawing section corresponding to the SO161-44 seismic stacking section modified from Fig. 3 in^94^, in which the main geological features and the seismic reflector indicating the transition between gas hydrate and free gas (e.g., the bottom simulating reflector, BSR) are indicated. In the inset, the location map showing static model location reported in Fig. 8 (red dashed line), and mud growing zone (red circle) and the SO161-44 position (black line).

To understand the depth stability of gas hydrate, the theoretical base of the GHSZ was calculated assuming a geothermal gradient of 30 °C/km (as per^25^), and a mixture of 95% methane and 5% ethane as per measures obtained at Ocean Drilling Program Site 1235^114^. The theoretical base of the GHSZ was calculated as the intersection between the hydrate stability curve and the temperature/pressure curve in the sediments (e.g.^115^). The first curve was evaluated by using the Sloan equations^17^, used to model a mixture of gases in freshwater^116^, equations were used to shift the freshwater hydrate curve because of water salinity effect^28^, 3.5% in our case. The second curve was evaluated considering water density of 1040 kg/m^3^, as per^28^. It is crucial to note that in our study area, hydrate and free gas data were detected by seismic analysis, confirming the site is characterised by upward fluid flow^25,93^. Figure 7 shows the main geological features, including the seismic indicator of the transition between the gas hydrate and the free gas zones, i.e. bottom simulating reflector (BSR), detected in a seismic line located near the relief.

**Figure 8:**
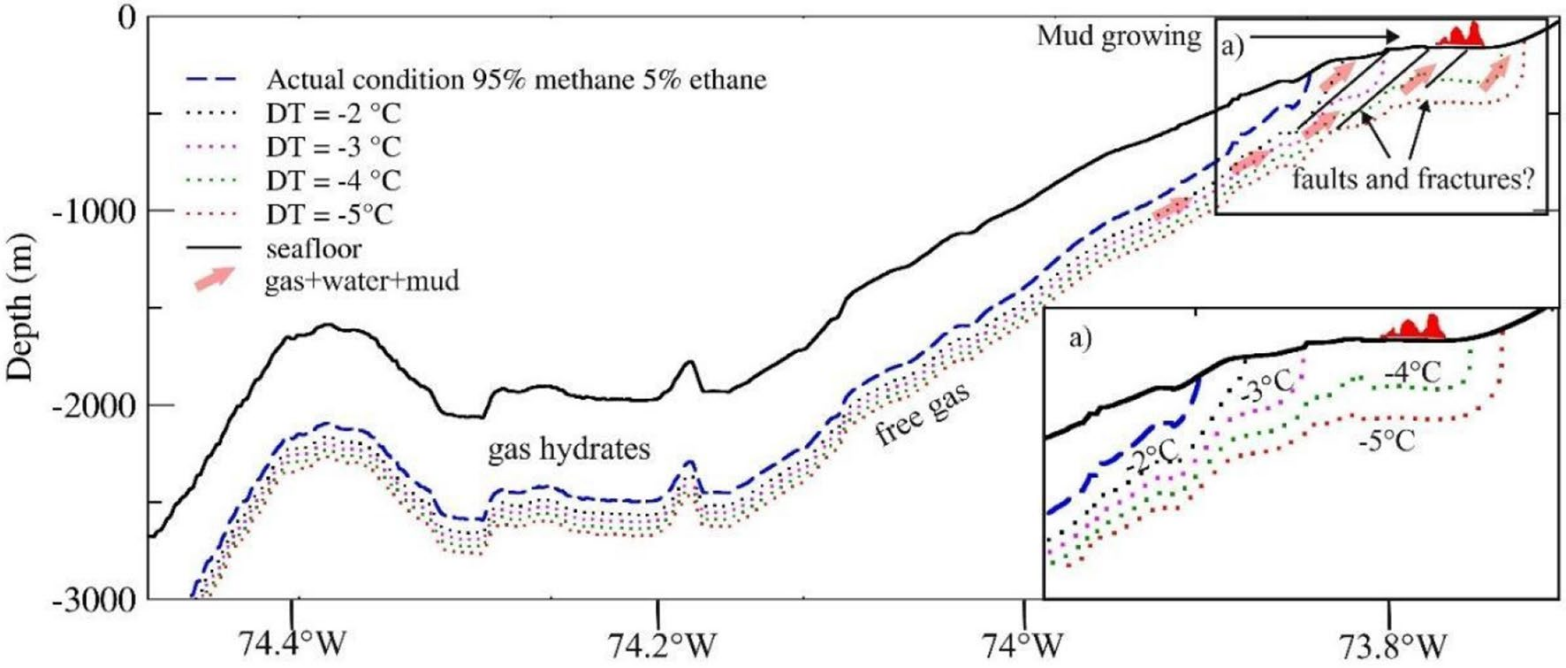
Schematic profile explaining mud growing formation (in red). The profile location is shown in Fig. 1. Dashed lines show theoretical bases of GHSZ by using the geothermal gradient of 30 °C/km for several scenarios supposing that the hydrate is formed by a mixture of 95% methane and 5% ethane. The blue dashed line indicates the actual theoretical base of the GHSZ. The dotted lines indicate the theoretical base of GHSZ supposing a decrease of the bottom temperature of 2 °C (black dotted line), 3 °C (magenta dotted line), 4 °C (green dotted line) and 5 °C (red dotted line). The solid black line indicates the seafloor. The pink arrows indicate the direction of the fluid/mud outflow. Possible faults and fractures are also reported as black lines. The rectangle in (a) shows the zoom, in which the modelling for the theoretical base of GHSZ reaches the continental shelf.

Figure 8 depicts the base of GHSZ at about 400 m water depth, indicating that at shallower depth, hydrate is not stable, and only free gas is present. Our study area is located in a narrow continental shelf (15 km width), favouring fluids advection associated with gas hydrate dissociation; thus, gas accumulations migrate from the base of GHSZ to shallow areas.

At higher latitudes, a substantial reduction of the GHSZ was observed due to warming in the last 20,000 years (e.g.^33,117,118^). To verify such trend in our study area, we modelled the theoretical base of the GHSZ, assuming past temperature conditions as reported by paleoclimate reconstruction studies^117,119^, e.g. decrease of seabed temperature by 1 °C, 2 °C, 3 °C, 4 °C, and 5 °C (Fig. 8). As per^117^, the modelled temperatures were characteristic of about 11,000, 12,000, 16,000, 17,000 and 18,000 yr BP respectively. It was assumed that other parameters necessary to assess hydrate stability (i.e. geothermal gradient, water depth, gas composition) remained invariant. The model indicated that the origin of the mud structures observed in this study may be related to hydrate dissociation caused by past increase of seabed temperature in around 12,000 yr BP. Additional measurements are necessary to test our hypothesis.

Alessandrini et al.^113^ recently acknowledge that this region of the Chilean margin may be critically disturbed in the long term, because of the tectonic-sedimentary configuration. Their model indicated that in the next 100 years, about 6.5% of the area where the gas hydrate is stable possibly will be affected by hydrate dissociation, induced by global warming. Hydrate dissociation may affect the Chilean margin seafloor morphology that is located close to the shoreline (less than 10 km)^113^.

## Conclusions

The positive relief indicated in our multidisciplinary study may be associated to mud cones by fluid-flux supply, which may be channelized through faults and fractures, as detected by seismic data^93^. δ^18^O and δD-enrichment of pore water, resulting from gas-hydrate melting and dissociation, actively support this observation. We suggest that the dissociation of gas hydrates, by tectonic uplift (i.e. decreasing pressure) and/or climate change (i.e. increasing temperature), generates a mixture of water enriched with heavy isotopes, mud, and gas, which may be expelled through the multiple faults present in our study area offshore Lebu. The discharge seems to be intermittent in time, like pulses, supporting the particular morphology of the monticules observed in the area. The region undergoes rapid hydrate dissociation, generating substantial carbon fluxes in short period (decades), as reported in other shallow areas, such as the Gulf of Mexico and Arctic (i.e.^120^). Sediment-grain size analysis provided information about (i) the type of sedimentary material that migrates from deeper to shallower zones, (ii) the anomalies of heavy isotopes concentrated in pore water, and (iii) the ideal environment sustaining chemosynthetic benthic organisms in this area, such as the foraminiferal genera *Bolivina*, *Uvigerina*, and found in shallow sediments.

Our research provides new information about gas-hydrates dissociation in shallow seabed features, i.e. mud cones on the continental shelf. It is critical to emphasise that fluid escapes in shelf seas may spread to the atmosphere releasing methane, thus contributing to global warming (e.g.^121^). Our results demonstrate that Chilean margin offshore Lebu is a suitable region for investigating such processes.
